# How to manage accidental and unintended exposure in radiology: an ESR white paper

**DOI:** 10.1186/s13244-019-0691-0

**Published:** 2019-02-21

**Authors:** European Society of Radiology (ESR)

**Affiliations:** Vienna, Austria

**Keywords:** Overexposure, Radiation, Incident, Accident, Reporting criteria, Significant event

## Abstract

Since February 2018, the Directive 2013/59/EURATOM (EU-BSS) requires all EU member states to implement a system for recording and analysis of all accidental or unintended medical exposures (Article 63). An ESR questionnaire in May 2018 among ESR member countries including all EU member states (MS) revealed a very heterogeneous and unsatisfactory situation in transposition of the EU-BSS. Some MS just translated this part of the directive, others used effective dose as reporting criteria and others used physical dose parameters from the modalities. This white paper will help national scientific organisations advice their national regulators and authorities on how to provide a simple and practicable implementation of the directive. ESR recommends notification and reporting criteria for significant events based on physical quantities and units and not on effective dose or text-based criteria like “significantly different” (EU-BSS, Article 4 (99)).

## Key points

- According to Directive 2013/59/EURATOM “significant dose events” require analysis and reporting to competent authorities.
- ESR recommends reporting physical dose parameters from dedicated modalities.
- These parameters are available as part of DICOM headers or Radiation Dose Structured Report (RDSR).

## Introduction

Radiation protection is one of the important scientific fields concerning the safety of patients in diagnostic and interventional radiology. The three fundamental principles of radiation protection are justification, optimisation and the application of dose limits (ICRP103) [1]. Under the umbrella of the EuroSafe Imaging Campaign [2], the European Society of Radiology (ESR) makes a strong commitment to all aspects of radiation protection of patients and occupational exposure of staff and the general population. A challenging task for European member states is the transposition of Council Directive 2013/59/EURATOM (EU-BSS) [3] requirements in the medical sector into national law. The ESR successfully evaluated the activities in transposition with an EC tender project [4]. Articles 63 and 96 of the EU-BSS are focussed on new requirements to recognise, manage and report accidental and unintended exposures. Article 63 (c) states:

for all medical exposures the undertaking implements an appropriate system for the record keeping and analysis of events involving or potentially involving accidental or unintended medical exposures, commensurate with the radiological risk posed by the practice

This is a new challenge as many EU member states (EU MS) have neither definitions of accidental and unintended exposures nor recognition or reporting criteria.

This paper is intended to help ESR institutional member societies, and in particular EU MS, in communicating with their regulators to find practicable solutions to implement these specific aspects of the directive.

## Exposure and justification

The principal justification of medical exposures is to do more good than harm to the patient. Medical exposures are unavoidable since ionising radiation is used in many procedures to diagnose or treat patients, providing benefits usually larger than the risk associated. The responsibility for the justification of the use of a particular procedure falls on the relevant medical practitioners [1, 5, 6]. Justification applies at three levels in the use of radiation in medicine.

- At level I, the use of ionising radiation in medicine is generally accepted as doing more good than harm to the patient.
- At level II, a specified procedure with a specified objective is defined and justified. The aim of level II justification is to judge whether the radiological procedure will usually improve the diagnosis or treatment of patients with similar clinical conditions. For example, an abdominal CT is justified at level II for patients showing symptoms of an aortic aneurysm with rupture, while it is not justified for a child with focal liver lesions. Level II justifications are published in national and international referral guidelines and should be evidence-based [7]. An abdominal CT of a child with focal liver lesions would not be justified at level II.
- At level III, the application of a specific procedure to an individual patient should be justified, taking into account the specific objectives of the planned exposure and clinical conditions of an individual patient. Interventional procedures for example involve the risk of unpredictable complexity with dose levels higher than intended. Therefore, if the radiation risk associated with a specific level III procedure (individual patient) exceeds the expected benefit of an examination or intervention, it is not justified.

From the point of view of radiation protection, unjustified examinations should be avoided, but not necessarily be understood as accidental or unintended overexposure in the sense of the EU-BSS.

## Exposure and optimisation

ICRP defines optimisation as the process of determining what level of protection and safety makes exposures, and the probability and magnitude of potential exposures, “as low as reasonably achievable” (ALARA), economic and societal factors being taken into account. Optimisation applies to all level II justifications and to all individual patients after level III justification. This means the type of procedure, the radiation dose, the other physical imaging parameters and the use of contrast media and other drugs must be adapted to the individual specific clinical question. As an example, for CT examinations, the use of appropriate scan length, number of scan series, dose modulation and iterative reconstruction are typical optimisation tools. To support the process of optimisation, the EU-BSS requires the establishment and implementation of “diagnostic reference levels” (DRLs) for all EU MS. The ESR supports the use of harmonised European DRLs with different research projects. For imaging of children, the PiDRL project [8] established age- and weight-dependant DRLs for common paediatric procedures. Today, most DRLs are based on anatomical regions or body parts to be examined but only few on clinical questions. The ongoing ESR EUCLID [9] project aims to establish clinical DRLs for adults where the radiation dose of a procedure for an anatomical region should be modified depending on the clinical question. For example, CT scans with standard abdominal parameters instead of a low-dose protocol for detection of ureteral stones would be non-justified overexposures [10].

## Significant dose events

Too high exposures of patients undergoing a specific procedure, imaging of a wrong body part or imaging the wrong patient are rare but may happen everywhere at any time. The term “significant events” is a summary of the definition given by the EU-BSS for unintended and accidental overexposures in Article 4 (99):

medical exposure that is significantly different from the medical exposure intended for a given purpose.

In case of diagnostic and interventional radiology, this is related to unnecessary overexposures of individual patients with the risk of deterministic effects, high effective doses or high organ doses (individual approach) or a group of patients with the risk of stochastic effects (collective approach). These two approaches were discussed at a Heads of the European Radiological protection Competent Authorities (HERCA) Working Group “Medical Applications” meeting, in Paris 2016. The collective approach involves exposure of a larger number of patients with low doses well below the threshold of deterministic effects. Reasons may be technical errors of a modality or human errors in the application of examination protocols or the use of the equipment. Chest radiograms of many patients over several days with missing aluminium/copper filter or incorrect antiscatter grid position could be an unintended collective overexposure if dose criteria for “significant” are met.

The individual approach involves exposure of one patient to doses that result in deterministic effects (such as skin injuries) or high effective doses or organ doses. Reasons may be technical errors of a modality or human errors in the application of an examination protocol or the use of the equipment. The individual approach also involves exposing a wrong patient due to confusion or a wrong organ or body side (e.g. wrong arm or leg). Interventional procedures with too many imaging series or excessive fluoroscopy time without changing the X-ray skin entrance field would be an unintended individual overexposure.

Radiation protection and patient safety require all efforts to prevent such incidents. If they occur, the first step should be in any case a local workup involving the practitioner, staff members, medical physics experts and radiation protection officer/expert. Referrer and patient should be informed about the incident. The definition of incidents may include near misses where an error was detected before performing a procedure with patient exposure. A summary of all possible errors, their causes and consequences for avoiding repetition can be found in [11].

## Reporting criteria of significant events

In Article 63 (e) (i) and Article 96, the EU-BSS requires a report to national authorities if an overexposure is classified as significant.

### Article 63 (e) (i)

the undertaking declares as soon as possible to the competent authority the occurrence of significant events as defined by the competent authority;

### Article 96: Notification and recording of significant events

Member States shall require the undertaking to:

(a) implement, as appropriate, a recording and analysis system of significant events involving or potentially involving accidental or unintended exposures;

(b) promptly notify the competent authority of the occurrence of any significant event resulting or liable to result in the exposure of an individual beyond the operational limits or conditions of operation specified in authorising requirements with regard to occupational or public exposure or as defined by the competent authority for medical exposure, including the results of the investigation and the corrective measures to avoid such events

However, the EU-BSS leaves the definition of “significant events” to the implementation of EU MS leading to confusion and a very heterogeneous approach across Europe. On April 4, 2018, a questionnaire on the implementation of EU-BSS Articles 63 and 96 was sent to all 47 ESR national societies including the societies in 28 EU MS. The deadline was June 1, 2018. Responses came from 19 of 28 EU MS (68%) and from 9 of 19 non-EU MS (47%). The data and evaluations in Table 1 and the examples in Appendix refer only to the responding EU MS.

**Table 1:** Summary of ESR questionnaire results on implementation of EU-BSS Articles 63 and 96, definition of “significant events”, reporting criteria in diagnostic and interventional radiology and to whom to report. The results include 19 of 28 (68%) responding EU MS

Implementation of Articles 63 and 96 into national law	10/19 (53%)
Definition of “significant event”	8/19 (42%)
Reporting of significant event to:	
- National authorities	14/19 (74%)
- Local or no answer	4/19 (21%)
Reporting criteria in diagnostic radiology:	
- P_KA_, CTDIvol, DLP, ESAK, AGD	7/19 (37%)
- Effective dose	2/19 (11%)
- Others (text based)	8/19 (42%)
Reporting criteria in interventional radiology:	
- P_KA_, CTDIvol, DLP, ESAK	9/19 (47%)
- Effective dose	1/19 (5%)
- Others (text based)	7/19 (37%)
Reporting of near misses or wrong patient or wrong body part	
- Near misses local, exposed patients national	5/19 (26%)
- All national	4/19 (21%)
- All local	2/19 (11%)
- No definition or requirement	7/19 (37%)

The results reveal the difficulties in finding a harmonised approach with about 50% of all responders having no precise definition of what “significant” means and no physical reporting criteria. Table 1 summarises the results of 19 responding EU MS.

## Dose management

Dose management is a common term, including dose recording, dose reporting, dose tracking and dose monitoring. Table 2 gives short definitions and applications of tasks related to dose management and analysis.

**Table 2:** List of different tasks under the umbrella of “dose management”

• Dose recording: part of clinical record, mandated by regulators, storage: PACS, RIS, paper-based, electronic recording required by EU-BSS Art. 60 1. (3) for CT and interventional radiology • Dose reporting: part of report for individual patients (2013/59/EURATOM), mandated by authorities to establish DRLs • Dose tracking: individual tracking of patient dose, patient safety, QA • Dose monitoring: QA, benchmarking, dose alerts (national DRLs, Local DRL’s, overexposure, accident reporting), technical malfunction, errors of staff • Dose management: includes all of above, scientific or industrial software solutions, needed for PACS, RIS, HIS, DICOM, IHE, dose management software	

About five years ago, some manufacturers started to offer software systems for dose management. These systems receive dose and other technical or patient parameters from individual modalities (CR, computed radiography; DX, digital radiography; CT, computed tomography; RF, radio fluoroscopy; MG, mammography; XA, X-ray angiography) either through analysis of DICOM headers or Radiation Dose Structured Reports (RDSR). Analysis of these parameters over a longer time axis provides data that are helpful in terms of radiation protection, optimisation, quality management, exposure analysis of individual patients and reporting of specific events. Commercial software systems are often very powerful in analysing dose parameters from many modalities and offered at high prices. It should be noted critically that in most systems, contrary to the recommendations of the ICRP [12], attempts are also made to calculate effective doses for individual patients or dose tracking in terms of ED over a longer time scale. Dose tracking may help to identify patients who have received too many potentially unjustified examinations over a longer time period, but this should not lead to justified procedures being rejected.

Such dose management software systems can be very helpful in the sense of implementing Articles 63 and 96 of the EU-BSS, but they are not necessarily, as often erroneously claimed, mandatory. If national reporting criteria are based on physical exposure parameters, they can easily be implemented into the dose management software after creating appropriate dose tables as trigger levels. This requirement, however, is met in only a few EU MS so far. In case of CT, a rough patient dose estimate is already available before starting the scan by a chosen CTDIvol for a reference body or head phantom (32 or 16 cm diameter). During patient exposure, the CTDIvol is modified more or less by *x*/*y* and *z* dose modulation. Hence, before starting a CT scan, the vendors could set up and display more realistic alert or notification levels than today.

## ESR recommendations

Dose parameters of a specific diagnostic procedure are first of all seen or registered by a radiographer, recognising too high exposures or exceeding of certain threshold values. The dose parameters of modalities are always based on physical units and quantities in the header of DICOM images or in the DICOM RDSR. DRLs which are applicable in most countries worldwide and in all EU MS are also based on these quantities (dose area product = P_KA_, CT Dose Index = CTDIvol, dose length product = DLP, entrance air kerma = Ka,r, average glandular dose = AGD).

From a practical point of view, ESR recommends notification and reporting criteria for significant events based on physical quantities and units and not on effective dose or text-based criteria like “significantly different”.

Appendix shows that some EU MS use reporting criteria based on DRLs. DRL trigger levels are exposure parameters of a specific procedure exceeding a DRL by a multiplication factor (typically factor 2–5). In Appendix c, Germany uses trigger levels for the collective approach. If a single procedure exceeds the DRL by 200%, the professional performing the study has to check whether the last 20 consecutive procedures exceeded the DRL by more than 100%. Beyond their use for reporting of significant events, trigger levels should be used to establish local notification or alert values [13]. The DRL multiplication factors could be used also to derive absolute dose values tables.

Some EU MS use reporting criteria based on effective dose (ED) values (mSv). The ESR does not recommend this approach as ED is not a dose output parameter of modalities and the radiologist has to convert the physical dose parameters more or less precisely by means of tables or algorithms into ED trigger levels. Furthermore, this approach involves several errors for individual patients. ED is an average over internal/external exposure, gender, age and dose rate. Hence, the ICRP recommends not using ED as a risk parameter for individual patients [12].

Concerning dose management software, the ESR encourages software vendors to develop affordable dose management systems that meet the basic requirements of the national reporting criteria while avoiding unnecessary or not recommended features such as calculating effective doses or too complex dose tracking tools.

## Appendix

### Examples for reporting criteria from Ireland, UK and Germany

- Ireland MERU [14]
  Exposure much greater than intended, for example:
  - Diagnostic overexposure (including nuclear medicine) of an adult as a result of more than twice the exposure intended (* see example below) that leads to an overexposure of > 10 mSv or 20 times the dose intended, regardless of the dose level
  - Diagnostic overexposure (including nuclear medicine) of a child as a result of more than twice the exposure intended that leads to an overexposure of > 3 mSv or 15 times the dose intended, regardless of the dose level
  - Deterministic effects produced as a result of interventional radiology
  - Dose given to carers without consent that is greater than medical council guidelines of 3 mSv and 15 mSv for adults 60 years or over
  Exposure where none intended, for example:
  - Dose to the breastfed child over 1 mSv
  - Inadvertent dose to foetus over 1 mSv
  - Incorrect patient (radiology, nuclear medicine or radiotherapy) exposed to over 1 mSv
- UK 2017 [15]
  **Table:**
  Diagnostic and interventional exposures	Guideline factor applied to intended dose
  High dose examinations, where the intended dose is greater than 5 mSv, to include interventional radiology, radiographic and fluoroscopic procedures involving contrast agents, diagnostic nuclear medicine, PET-CT and CT examinations	When the total exposure is at least 2.5 times greater than the intended dose
  Intermediate dose examinations, where the intended dose is within the range 0.5–5 mSv, to include mammography, CT scout examinations and all other radiographic examinations not referred to elsewhere in this table	When the total exposure is at least 10 times greater than the intended dose
  Low-dose examinations, where the intended dose is less than 0.5 mSv, to include DEXA, skull, dentition, chest, in-vitro nuclear medicine	When the total exposure is at least 20 times greater than the intended dose

c) Germany BMU and BfS (draft under consultation, to be published Dec. 2018) [16]
  I. Reporting criteria of diagnostic procedures
    - Collective approach
      - Any exceeding of DRLs by more than 100% over 20 consecutive procedures if a single procedure exceeds the DRL by 200% (without radiography and dental CBCT)
    - Individual approach
      b) CT of the brain: CTDIvol > 120 mGy, CT of all other body parts: CTDIvol > 80 mGy
        Fluoroscopy and angiography: P_KA_ > 200 Gy × cm^2^
      c) Any repetition of a procedure due to technical errors or a patient/side confusion, if for the additional exposure criterion b) is met.
      d) Any occurrence of an unexpected deterministic effect
  II. Reporting criteria of interventional procedures:
    - Collective approach
      e) Any exceeding of DRLs by more than 100% over 20 consecutive procedures if a single procedure exceeds the DRL by 200%
      f) Any repetition due to technical errors or patient/side confusion if b) is met.
      g) Any occurrence of an unexpected deterministic effect
    - Individual approach
      h) P_KA_ > 500 Gy × cm^2^ if skin injuries grade II or higher occur within 21 days
      i) Any repetition due to technical errors or patient/side confusion
      j) Any occurrence of an unexpected deterministic effect

## Abbreviations

AGD: Average glandular dose
ALARA: As low as reasonably achievable
CTDI: Computed tomography dose index
DICOM: Digital Image Communication in Medicine
DLP: Dose length product
DRL: Diagnostic reference level
ESAK: Entrance surface air kerma
EU-BSS: European Basic Safety Standards (Directive 2013/59/EURATOM)
EUCLID: European Study on Clinical Diagnostic Reference Levels for X-ray Medical Imaging
HERCA: Heads of the European Radiological protection Competent Authorities
HIS: Hospital information system
ICRP: International Commission on Radiological Protection
IHE: Integrating the Healthcare Enterprise
MS: Member states
PACS: Picture archiving and communication system
PiDRL: European Diagnostic Reference Levels for Paediatric Imaging
P_KA_: Air kerma-area product
QA: Quality assurance
RDSR: Radiation Dose Structured Report
RIS: Radiological information system
